# Effect of maternal employment on exclusive breastfeeding practice among mothers of infants 6–12 months old in Wolkite town, Ethiopia: a comparative cross-sectional study

**DOI:** 10.1186/s12905-022-01816-9

**Published:** 2022-06-11

**Authors:** Amare Zewdie, Temesgen Taye, Abebaw Wasie Kasahun, Abdu Oumer

**Affiliations:** Department of Public Health, College of Medicine and Health Sciences, Wolkite University, Wolkite, Ethiopia

**Keywords:** Exclusive breastfeeding practice, Mother’s employment, Infant feeding, Comparative cross-sectional, Ethiopia

## Abstract

**Introduction:**

Exclusive breastfeeding (EBF) means providing only breast milk for infants for up to six months without the addition of solid or liquid matter. Even though EBF had great benefits for infants and mothers, the rate of EBF is so limited below the global target. In Ethiopia, the overall EBF practice is 59%. This low EBF practice had a great unexplained variation among employed and unemployed mothers. Therefore, this study aimed to compare EBF practice and associated factors among employed and unemployed mothers of infants aged 6–12 months in Wolkite town, Southern Ethiopia, 2020.

**Methods:**

A community-based comparative cross-sectional study was conducted in March 2020. A total sample of 485 (241 employed and 244 unemployed) study subjects was involved in the study. A simple random sampling technique was used to recruit study subjects. A pre-tested structured interviewer-administered questionnaire was used. Multivariable logistic regression was used to identify associated factors of EBF practice for the whole study participants and then for employed and unemployed mothers independently.

**Results:**

The pooled prevalence of exclusive breastfeeding practice was 63.9% [95% CI (59.8–68.2%)]. Exclusive breastfeeding practice was 54.8% [95% CI (48.5–61.4%)] and 73% [95% CI (66.8–78.7%)] among employed and unemployed mothers respectively. Three or more years of a birth interval [AOR = 4.03; 95% CI (1.80–8.99)], three or more ANC visits [AOR = 5.39; 95% CI (1.49–19.45)], and having PNC service [AOR = 4.56; 95% CI (2.0–9.4)] significantly associated to exclusive breastfeeding practice among employed mothers. No history of breastfeeding counseling during ANC visits [AOR = 0.15; 95% CI (0.06–0.41)], had history of breast disease [AOR = 0.28; 95% CI (0.08–0.99)], three or more ANC visits [AOR = 5.11; 95% CI (1.66–15.8)], and having social support [AOR = 3.05; 95% CI (1.23–7.6)] significantly associated to EBF practice among unemployed mothers.

**Conclusion:**

Employment among mothers was found to discourage EBF practice. The predictors of exclusive breastfeeding practice are different for employed and unemployed. Therefore Policymakers and program planners are called to come together and create a conducive environment for lactating employees, and appropriate intervention at respective predictor variables is needed to enhance EBF practice.

## Introduction

Exclusive breastfeeding (EBF) means providing only breast milk for infants for up to six months without the addition of solid or liquid matter with the exception of oral rehydration solution, or drops/syrups of vitamins, minerals, or medicines [1, 2]. Breast milk in the first six months contains all the necessary nutrients provided in a bioavailable and easily digestible form. It is among the most effective ways to promote maternal and child health [3]. Exclusively breastfed infants can be protected from diarrhea, acute respiratory infections, risk of obesity, and allergies [4]. It is also important to the mother in returning the uterus to pre-pregnancy size faster; reduces the risk of breast, ovarian, uterine cancers, and osteoporosis. It also creates a special bond between mother and infant and aids in household food security by saving money [5, 6]. World health organization recommends EBF for the first six months of life. Then safe and adequate foods should be started after the six months, and breastfeeding continued up to two years and beyond [1, 7].

Globally, in 2021 only 44% of infants 0–6 months old are exclusively breastfed [8]. In Sub-Saharan Africa and East Africa, EBF practice was 31% and 42% respectively [9]. Ethiopian demographic health survey (EDHS) 2019 showed that EBF practice in Ethiopia was estimated to be 59% [10]. Despite appropriate feeding practice, being the most cost-effective intervention to reduce child morbidity and mortality, WHO in 2013 reported that non-exclusive breastfeeding contributes to 11.6% of mortality in children under the age of five years. This was equivalent to about 804,000 child deaths [11]. UNICEF in 2018 noted that improving breastfeeding rates around the world could save the lives of more than 820,000 children under the age of five every year [3]. Non-exclusive breastfeeding results in an estimated 40% of under‐five stunting in Western and Central Africa [12]. In Ethiopia, adequate EBF practices are expected to save the life of 70,000 infant deaths per year which is 24% of the total infant deaths annually [13].

In 2016, Ethiopia developed several nutritional strategies to increase the nutritional status of the child. These are: Promoting EBF for the first 6 months, establishing a baby-friendly health facility initiative in all public and private health facilities, enforcing the International Code of Marketing for Breast milk Substitutes, promoting the enactment of maternity leave, implementing breastfeeding rooms in major service providing institutions, and support employed breastfeed mothers to exclusively breastfeed their child [14]. Although repeated recommendations were given to EBF practice in Ethiopia, a trend study from 200 to 2016 revealed that the improvement in EBF practice was not statistically significant [15]. Similarly, EDHS showed that EBF practice had no significant improvement from 2016 to 2019 since only one percent has been increased [10].

Several factors affect the EBF practice. Among these maternal employment, parity, maternal perception of the quantity of breast milk, maternal education, antenatal care visits (ANC), postnatal care (PNC) utilization, maternal health, and birthplace are the most frequently associated factors [16]. Concerning maternal employment, there is a great significant variation in EBF practice among employed and unemployed mothers [17–19]. Despite women's labor forces being increased at an alarming rate in Ethiopia, EBF practice among employed mothers is relatively lower than among unemployed mothers [17–19]. The reason for the wide observed difference in EBF practice among employed and unemployed women is not well explained unless a few hypotheses that explain early resumption to work will reduce EBF practice among employed mothers. The aforementioned studies were done when the post-partum maternity leave was three months. Even though no a day’s post-partum maternity leave is increased from three to four months as a national nutritional strategy, EBF practice after the policy change is not known. Therefore, this study aimed to assess the association between maternal employment and EBF practices among mothers with infants between the ages of 6 and 12 months.

The study provides important evidence inputs for designing programs to improve EBF practice by considering the factors that affect the EBF practice of mothers in each group. The result of this study also has a potential contribution for program planners to design strategies to support employed breastfeeding mothers; such as implementing a breastfeeding room program in their working area. Considering those and similar rationale this study intended to assess the level of exclusive breastfeeding practice and associated factors among employed and unemployed mothers with an infant aged 6–12 months in Wolkite town, Southern Ethiopia, 2020.

## Method and materials

### Study design, period, and setting

A community-based comparative cross-sectional study design was conducted from February 20 to March 28, 2020, at Wolkite town which is found 158 km away from Addis Ababa, the capital city of Ethiopia in the southwest direction along the main road from Addis Ababa to Jimma. Wolkite town administration is organized into 3 sub-city and 7 kebeles. According to the Wolkite town administration health office report of 2019, the population size of the town is estimated to be 72,929, from this 15,413 are estimated to be 15–49 years old women and 1,106 are infants between 6 and 12 months of age [20].

### Population and sample

All mothers who had infants 6–12 months old (employed and unemployed mothers) and who live in the study area for at least six months were the source population. Mothers who had infants between 6 and 12 months old from simple randomly selected households were the study population. Mothers of babies with cleft lip, and/or cleft palate and severely ill mothers were excluded.

The sample size was determined for the two objectives (prevalence and associated factors).

The sample size for the first objective.

According to a previous study conducted in the Fafan zone, Somali regional state of Ethiopia in 2019, the prevalence of EBF among employed mothers was 25%, and the prevalence of EBF among unemployed mothers was 83% [17]. Then the sample size is determined by using the following statistical formula.

$$n = \left( {p1q1 + p2q2} \right) \left( {f\left( , \right)} \right) / (\left( {p1 - p2} \right)^{2}$$

$${\text{n}} = \frac{{\left( {0.25 \times 0.75 + 0.83 \times 0.17} \right) \times 7.84 \, }}{{\left( {0.25 - 0.83} \right)^{2} }} = 8,\;{\text{for}}\;{\text{each}}\;{\text{employed}}\;{\text{and}}\;{\text{unemployed}}\;{\text{mothers}}$$

where P1 = 0.25 (prevalence of EBF among employed), q1 = 1 − p1, P2 = 0.83 (prevalence of EBF among unemployed), q2 = 1 − p2 and f (α, β) = 7.84; when the power = 80% and α = 5%.

The sample size for associated factors is calculated using a comparative study done at Gonder town with the assumptions of 95% confidence interval, 80% power using OpenEpi version 3.01 [19] presented as follows (Table 1).

**Table 1:** Sample size calculation for the study by considering associated factors Wolkite, 2020

Outcome variable	Associated factors	CI	Power	Ratio	AOR	Outcome among		Total sample
						Exposed	Unexposed	
EBF practice	Place of birth	95	80	1	0.53	239	239	478
	Knowledge of EBF practice	95	80	1	0.437	158	158	316
	Marital status	95	80	1	1.88	205	205	410
	Employment status	95	80	1	3.43	56	56	112

Finally, the calculated sample sizes by using the first objective and associated factors were compared. Then the largest sample size was taken. Therefore, the largest sample size of 239 in each group was taken, and after considering of 5% non-response rates the total sample size for each group was 251,

Regarding the sampling procedure first, the sample size was proportionally allocated to each kebele based on the total number of targeted women-child pairs in each kebele. Then household survey was conducted before data collection to identify the employment status of the mother and to provide an identity code for each eligible household in all kebeles. After having the number of employed and unemployed mothers, again the total sample size was proportionally allocated by employment status in each kebele. Then computer-generated simple random sampling technique was used to select study participants.

### Data collection instrument and procedure

Data were collected by face-to-face interviews using pre-tested structured questionnaires which were prepared after reviewing different literature such as EDHS,2016 questionnaires, and other published literature on related topics [17–19, 21–24]. The questionnaire consists of five parts; socio-demographic characteristics, maternal health service and infant related factors, breastfeeding and related items, maternal breastfeeding-related knowledge, and other barriers to exclusive breastfeeding. Data were collected by four clinical nurse professionals with the supervision of two public health professionals.

### Study variables

Exclusive breastfeeding practice was the dependent variable. Independent variables include; socio-demographic factors such as maternal age, educational level, employment status, knowledge, working hours, paternal educational level, paternal occupation, Family size, household income, and social support. Maternal health-related factors including parity, number of ANC visits, breastfeeding counseling during pregnancy, Place, and mode of delivery, postnatal care, maternal illness, and Perceived adequacy of breast milk were predictor variables. Similarly, infant-related factors such as birth order and interval, Sex and health status of the infants, and Bottle feeding practice were independent variables.

### Operational definitions

An employed mother is a mother who had employed at a governmental or private organization or day laborer who works for more than eight hours per day for at least five days per week, and who had employed from birth to six months of the eligible child age.

*Unemployed mother*: a mother, who works at her home as a housewife,

*Social support*: a woman who had given economic, psychological support, and/or breastfeeding counseling from the society or organization.

*Maternal breastfeeding knowledge*: There are ten maternal knowledge-related questions, and if a study participant responds to five and above correct answers, she has good knowledge and if she responds to less than five, she has poor knowledge [22, 25].

### Data quality assurance

To ensure the quality of data, three days of training were provided to data collectors and supervisors.

To assess the appropriateness of wording, clarity of the questions, and respondent reaction to the questions and interviewer, a pre-test was done on 5% of the calculated sample size in Emdibir town which has socio-demographic similarities with our study population. Regular supervisions were made during data collection. The collected data were checked for completeness, consistency, and clarity on daily basis.

### Data analysis

The data were coded and entered into EpiData version 3.12 statistical software and then exported to SPSS version 21 for further analysis. Descriptive statistics such as frequencies, mean, median, and proportions were computed to find out the prevalence of exclusive breastfeeding (EBF) and other indices. Bivariate logistic regression was used to check variables having an association with the dependent variable, and then those variables having a p-value of ≤ 0.2 were fitted to multivariable logistic regression for controlling the effects of confounders. Adjusted Odds Ratios with 95% CI and a *P*-value of < 0.05 in multivariable logistic regression models were used to declare a significant association of variables with the dependent variable. Three multivariable analysis models were fitted; first, the model was fitted for the whole study participants. Then the model was fitted separately for employed and unemployed study subjects.

## Results

### Socio-demographic characteristics

In this study, a total of 241 (49.7%) employed and 244 (50.3%) unemployed women participated with a cumulative response rate of 97%. Concerning age category 92 (38.2%) of employed and 87 (35.7%) of mothers are in the age group of 25–29 years. Most 211 (88%) of employed and 222 (91%) of unemployed mothers were married. Majority189 (78.4%) of employed mothers had a college education and above, but 22 (8%) of unemployed mothers had college and above educational level (Table 2).

**Table 2:** Socio-demographic characteristics of mothers who have infants aged 6 to 12 months, in Wolkite town, South, Ethiopia, 2020

Variables	Categories	Employment status of the mother					
		Employed (N = 241)		Unemployed (N = 244)		Overall (N = 485)	
		Frequency	Percent (%)	Frequency	Percent (%)	Frequency	Percent (%)
Maternal age in years	15–19	0	0	2	0.8	2	0.4
	20–24	36	14.9	52	21.3	88	18.1
	25–29	92	38.2	87	35.7	179	36.9
	30–34	77	32	64	26.2	141	29.1
	≥ 35	36	14.9	39	16	75	15.5
Religion	Muslim	116	48.1	119	48.88	235	48.5
	Orthodox Christian	81	33.6	72	29.5	153	31.5
	Catholic	12	5	9	3.7	21	4.3
	Protestant	31	12.9	42	17.2	73	15.1
	Others*	1	0.4	2	0.8	3	0.6
Ethnicity	Guraghe	127	52.7	123	50.4	250	51.5
	Kebena	40	16.6	44	18.0	84	17.3
	Amhara	38	15.8	35	14.3	73	15.1
	Oromo	14	5.8	15	6.1	29	6.0
	Others**	22	9.1	27	11.1	49	10.1
Maternal education	Can’t read and write	0	0	10	4.1	10	2.1
	Can read and write	3	1.2	18	7.4	21	4.3
	Primary education	14	5.8	75	30.7	89	18.4
	Secondary education	35	14.5	119	48.8	154	31.8
	Collage education and above	189	78.4	22	8.0	211	43.5
Marital status	Married	211	87.6	222	91.0	433	89.3
	Widowed	4	1.7	1	0.4	5	1.0
	Divorced	13	5.4	11	4.5	24	5.0
	Separated	13	5.4	10	4.1	23	4.7
Monthly household income in ETB	≤ 2000	28	11.6	71	29.1	99	20.4
	2001–3500	52	21,6	93	38.1	145	29.9
	> 3500	161	66.8	80	32.8	241	49.7

*No religion and Adventist

**Wolaita, Silte, Tigrie, Hadya, Gamo…

Among employed study participants, 155 (64%) mothers were governmentally employed.

The majority of husbands among employed mothers 123 (55%) had a college education and above. Whereas only 49 (21%) of husbands among unemployed mothers had a college education and above.

### Maternal health service and infant related characteristics

The study revealed that more than half 286 (59%) of mothers had one to two children. The majority of the employed study participant 232 (96.3%) had a history of at least one ANC service follow-up at a health facility throughout their pregnancy. The study showed that almost all 240 (99.6%) employed study participants and 241 (99.2%) unemployed study participants delivered at a health facility, but about 124 (51.5%) of employed and 107 (43.9%) unemployed study subjects had post-natal care service (Table 3).

**Table 3:** Maternal health service and infant related characteristics of mothers of infants aged 6–12 months in Wolkite town, Southwest Ethiopia, 2020

Variables	Categories	Maternal employment status					
		Employed (N = 241)		Unemployed (N = 244)		Overall (N = 485)	
		Frequency	Percent (%)	Frequency	Percent (%)	Frequency	Percent (%)
Number of children	1–2	147	61	139	57	286	59
	≥ 3	94	39	105	43	199	41
Sex of the infant	Male	116	48.1	126	51.6	242	49.9
	Female	125	51.9	118	48.4	243	50.1
Birth order of infant	1–2	149	61.8	142	58.2	291	60
	≥ 3	92	38.2	102	41.8	194	40
Birth interval in month	≤ 35	57	32.2	39	22	96	27
	≥ 36	120	67.8	138	78	258	73
ANC visit	Yes	232	96.3	243	99.6	475	98
	No	9	3.7	1	0.4	10	2
Number of ANC visits	1–2	32	13.8	33	13.6	65	13.7
	≥ 3	200	86.2	210	86.4	410	86.3
Breastfeeding counseling during ANC	Yes	186	77.2	193	79.4	379	79.8
	No	46	19.1	50	20.6	96	20.2
Place of delivery	Health facility	240	99.6	242	99.2	482	99
	Home	1	0.4	2	0.8	3	1
Mode of delivery	Normal/vaginal	225	93.4	232	95.1	457	94
	C/S	16	6.6	12	4.9	28	6
PNC service	Yes	124	51.5	107	43.9	231	47.6
	No	117	48.5	137	56.1	254	52.4
Breastfeeding counseling during PNC	Yes	120	96.8	104	97.2	224	97
	No	4	3.2	3	2.8	7	3
Social support for EBF	Yes	105	43.6	130	53.3	235	48.5
	No	136	56.4	114	46.7	250	51.5
Maternal EBF knowledge	Poor	16	6.6	8	3.3	24	5
	Adequate	225	93.4	236	96.7	461	95

### Prevalence of exclusive breastfeeding practice

The overall exclusive breastfeeding practice among the study participants was 63.9% (59.8–68.2%). The practice of exclusive breastfeeding among employed and unemployed mothers was 54.8% (95% CI 48.5–61.4%) and 73% (95% CI 66.8–78.7%) respectively**.**

In this study, the majority 236 (98%) of employed and 234 (96%) of unemployed mothers have fed the first breast milk or colostrum to their newborn. The most 26 (38.8%) frequently reported reason to discontinue exclusive breastfeeding among unemployed mothers was the perception of feeding breast milk only was not sufficient for the infant until six months. On the other hand, among 69 (63.3%) employed mothers the main reason to quit exclusive breastfeeding practice was lack of time.

This study also found that only 14 (5.8%) of employed mothers had breastfeeding rooms in their working areas, and 226 (94%) of them said that EBF is difficult to achieve at work. The study also revealed that about 180 (75%) of employed mothers had maternity leave with payments. Of those who had maternity leave with payment about 160 (97%) of them had 16 weeks and longer maternity leave.

### Factors associated with exclusive breastfeeding practice

#### Factors associated with exclusive breastfeeding practice among all participants

In multivariable regression mothers who had three or more ANC, visits were 6.33[AOR = 6.33; 95% CI (2.85–14.06)] times more likely to exclusively breastfeed their infant than mothers who had less than three ANC visits. Mothers who had postnatal care were 3.77[AOR = 3.77; 95% CI (2.16–6.59)] times more likely to practice exclusive breastfeeding than those mothers who had no postnatal care. Having birth intervals of three or more years were 3.18 times more likely to practice EBF than those who had less than three years’ birth intervals [AOR = 3.18; 95% CI (1.79- 5.65)]. Unemployed mothers were 2.97 times more likely to exclusively breastfed their infant compared to employed mothers [AOR = 2.97: 95% CI (1.71–5.17)] (Table 4).

**Table 4:** Bi-variable and multivariable analysis of factors associated with exclusive breastfeeding practice among mothers of infants aged 6–12 months in Wolkite town, Southwest Ethiopia, 2020

Variables	Categories	EBF practice for six months		COR (95% CI)	AOR (95% CI)	*P* value
		Yes	No			
Number of children	1–2	166 (53.5%)	120 (68.6%)	1	1	
	≥ 3	144 (46.5%)	55 (31.4%)	1.89 (1.28, 2.80)	1.34 (0.8, 2.36)	0.245
Birth interval in months	< 36	42 (43.8%)	54 (56.3%	1	1	
	≥ 36	194 (56.3%)	64 (24.8%)	3.99 (2.38, 6.38)	3.18 (1.79, 5.65)	< 0.0001
Number of ANC visit	1–2	19 (29.2%)	46 (70.8%)	1	1	
	** ≥ 3**	291 (70%)	119 (29%)	5.92 (3.33, 10.53)	6.33 (2.85, 14.06)	< 0.0001
Postnatal care	Yes	188 (81.4%)	43 (18.6%)	4.73 (3.13, 7.15)	3.77 (2.16, 6.59)	< 0.0001
	No	122 (48%)	132 (52%)	1	1	
Knowledge of EBF	Poor	4 (1.3%)	20 (11.4%)	1	1	
	Adequate	306 (98.7%)	155 (88.6%)	9.87 (3.32, 29.82)	1.63 (0.24, 11.23)	0.618
Having social support	Yes	181 (77%)	54 (23%)	3.14 (2.12, 4.65)	1.66 (0.97, 2.86)	0.067
	No	129 (51.6%)	121 (48.4%)	1	1	
History of breast disease	Yes	16 (6.1%)	26 (14.9%)	1	1	
	No	291 (93.9%)	149 (85.1%)	2.67 (1.43, 4.99)	2.01 (0.84, 4.83)	0.117
Employment status	Employed	131 (54.6%)	109 (45.4%)	1	1	
	Unemployed	179 (73.1%)	66 (26.9%)	2.26 (1.54, 3.3)	2.97 (1.71, 5.17)	< 0.0001

#### Factors associated with exclusive breastfeeding among employed mothers

Among employed mothers, a birth interval of three or more years was 4.03 more likely to practice EBF than those who had less than three years of a birth interval [AOR = 4.03; 95% CI (1.80–8.99)]. Employed mothers who had three or more ANC visits at health facilities were five times more likely to exclusively breastfeed their infant than those mothers who had less than three antenatal care visits [AOR = 5.39; 95% CI (1.49–19.45)]. Employed mothers who had postnatal care were 4.56 times more likely to practice EBF than those mothers who had no postnatal care [AOR = 4.56; 95% CI (2.0–9.4)] (Table 5).

**Table 5:** Bi-variable and multivariable analysis of factors associated with exclusive breastfeeding practice among employed mothers of infants aged 6–12 months in Wolkite town, Southwest Ethiopia, 2020

Variables	Categories	EBF practice for six months		COR (95% CI)	AOR (95% CI)	*P* value
		Yes	No			
Number of children	1–2	73 (55.3%)	74 (67.9%)	1	1	
	≥ 3	59 (44.7%)	35 (32.1%)	1.71 (1.01, 2.99)	1.02 (0.48, 2.17)	0.952
Birth interval	< 36 months	21 (20.6%)	36 (48%)	1	1	
	≥ 36 months	81 (79.4%)	38 (52%)	3.56 (1.84, 6.89)	4.03 (1.80, 8.99)	0.001
Number of ANC visits	1–2	6 (4.5%)	26 (26%)	1	1	
	≥ 3	126 (95.5%)	74 (74%)	7.4 (2.90, 18.76)	5.39 (1.49, 19.45)	0.01
Postnatal care	Yes	94 (71.2%)	30 (27.5%)	6.51 (3.70, 11.46)	4.56 (2.0, 9.4)	< 0.0001
	No	38 (28.8%)	79 (72.5%)	1	1	
Knowledge	Poor	3 (2.3%)	13 (11.9%)	0.17 (0.048, 0.62)	1.02 (0.1, 10.61)	0.986
	Adequate	129 (97.7%)	96 (88.1%)	1	1	
Social support	Yes	69 (52.3%)	36 (33%)	2.22 (1.31, 3.76)	1.28 (0.62, 2.65)	0.507
	No	63 (47.7%)	73 (67%)	1	1	
Presence of maternity leave	Yes	115 (87.8%)	65 (59.6%)	4.87 (2.55, 9.30)	2.45 (0.94, 6.4)	0.068
	No	16 (12.2%)	44 (40.4%)	1	1	

#### Factors associated with exclusive breastfeeding among unemployed mothers

In multivariable analysis, unemployed mothers who had three or more ANC visits to a health facility were 5.11 times more likely to practice EBF as compared to mothers who had less than three ANC visits [AOR = 5.11; 95% CI (1.66–15.8)]. Unemployed mothers who were not given breastfeeding counseling during ANC visits were 85% less likely to EBF than those unemployed mothers who had breastfeeding counseling during ANC visits [AOR = 0.15; 95% CI 0.15 (0.06–0.41)]. Unemployed mothers who had social support were 3.05 times more likely to practice EBF than those mothers who had no social support [AOR = 3.05; 95% CI (1.23–7.6)]. Similarly, mothers who had a history of breast disease were 72% times more likely to cease EBF compared to mothers who had no history of breast disease [AOR = 0.28; 95% CI (0.08–0.99)] (Table 6).

**Table 6:** Bi-variable and multivariable analysis of factors associated with exclusive breastfeeding practice among unemployed mothers of infants aged 6–12 months in Wolkite town, Southwest Ethiopia, 2020

Variables	Categories	EBF practice for six months		COR (95% CI)	AOR (95% CI)	*P* value
		Yes	No			
Number of children	1–2	93 (52.2%)	46 (69.7%)	1	1	
	≥ 3	85 (47.8%)	20 (30.3%)	2.1 (1.15, 3.84)	2.16 (0.9, 5.2)	0.085
Birth interval	< 36 months	21 (15.7%)	18 (41.9%)	1	1	
	≥ 36 months	113 (84.3%)	24 (58.1%)	3.87 (1.8, 8.32)	2.17 (0.82, 5.73)	0.120
Having breastfeeding counseling during ANC	Yes	155 (87%)	38 (58%)	1	1	
	No	23 (13%)	27 (42%)	0.21 (0.11, 0.4)	0.15 (0.06, 0.41)	< 0.0001
Number of ANC	1–2 times	13 (7.3%)	20 (30.8%)	1	1	
	≥ 3 times	165 (92.7%)	45 (69.2%)	5.64 (2.6, 12.21)	5.11 (1.66, 15.8)	0.005
PNC service	Yes	94 (52.8%)	13 (19.7%)	4.56 (2.33, 8.95)	2.00 (0.76, 5.25)	0.16
	No	84 (47.2%)	53 (80.3%)	1	1	
Knowledge of EBF	Poor	1 (0.6%)	7 (10.6%)	0.05 (0.01, 0.4)	0.125 (0.003, 5.99)	0.293
	Adequate	177 (99.4%)	59 (89.4%)	1	1	
Social support	Yes	112 (62.9%)	18 (27.3%)	4.53 (2.43, 8.42)	3.05 (1.23, 7.6)	0.016
	No	66 (37.1%)	48 (72.7%)	1	1	
History of breast disease	Yes	11 (6.2%)	13 (19.7%)	0.27 (0.11, 0.64)	0.28 (0.08, 0.99)	0.049
	No	167 (93.8%)	53 (80.3%)	1	1	

## Discussion

This study assessed the association between maternal employment status and exclusive breastfeeding practice among mothers of infants aged 6–12 months. Due to its several benefits for the child and the mother, WHO and UNICEF recommend exclusive breastfeeding for six consecutive months [1–3].

The prevalence of EBF in this study was 63.9% (59.8–68.2%) which is similar to the study done in different parts of Ethiopia such as; the Southern region 63.8%, Afar region 62%, Oromia region 65.6% [26], Hawassa 61% [27], Boditi Town, Woliata 64.8% [28] and with the study of global breastfeeding in twenty-first century 63% [29]. This finding is higher than the global target of exclusive breastfeeding practice of 50% in 2025 [7], Saudi Arabia 37% [30], African regions such as Zimbabwe 36% [31], Tanzania 38.8% [32], Kenya 45.5% [33], Ghana 50.6% [34], East Africa 42% [9], Somaliland 20.47% [35]. It is also higher than the mini Ethiopian demographic health survey,2019 report 59% [10], national trend studies in Ethiopia 59.9% [15], and Addis Ababa, Ethiopia 29.3% [36]. The potential variation of the finding of the current study from the two national studies might be due to study area difference since those studies use the national data which lower the prevalence while the current study focuses on a specific area (Wolkite town). The difference from the study in Addis Ababa might be the effect of the 2016 national nutritional strategy including the increment of maternity leave; since the study in Addis Ababa was done before the policy change. On the contrary, EBF practice in this study is lower than the study conducted in Bahirdar 86.4% [37]. The difference might be due to variation in measurement of exclusive breast feeding practice; since, the study in Bahirdar measures’ a one day pre survey EBF practice while the current study assess infant feeding during six months. The finding implies strong effort should be made to raise mother’s EBF practice including implementation of the national nutritional improvement strategy of 2016.

In this study, the prevalence of EBF among employed mothers was 54.8% (95% CI 48.5–61.4%) which is lower than EBF practice among unemployed mothers 73% (95% CI 66.8–78.7%). The finding is in line with the study conducted in Nepal [38], Ghana [23], and Kenya [24] and with studies done in Ethiopia at Injibara [18], Gondar [19], Debremarkos [22], and Somali region [17]. The significant difference might be due to the fact that employed mothers might not have adequate time to feed breast milk to their infants during working hours as unemployed mothers. Since, unemployed mothers usually have flexible working hours unlike employed mothers, which may contribute to relatively higher adherence to EBF practice than employed mothers.

Regarding factors associated with EBF, the study revealed that unemployed mothers were three times more likely to practice EBF than employed mothers. This finding is similar to the studies done in Tanzania [32], sub-Saharan countries [39], and the studies in Ethiopia such as; Debremarkos [22], Injibara [18], Bahirdar [37], Gondar [19], Hawassa [27] and Jigjiga [17]. The possible explanation might be unemployed women had more time to stay with their babies than employed mothers. In addition to this, lack of safe breastfeeding room at the workplace, less flexibility of managers for breastfeeding breaks, remoteness of working place from home, long working hours per day, and absence or short period of maternity leave might be the reasons for the decrement in EBF practice in employed mothers [38]. For instance, the presence of longer maternity leave was directly related to EBF practice compared with mothers who worked without maternity leave [40]. This finding suggests adequate support should be given to employed mothers including; permitting appropriate maternity leave so as to improve EBF practice.

Our study found that Mothers who had three or more ANC visits were 6.33 times more likely to practice EBF compared to women who had less than three ANC visits. This is consistent with the study on sub-Saharan countries [39]. This finding suggests adequate optimal breastfeeding counseling had been given to the mothers during frequent antenatal care visits since that is why EBF practice is higher in those mothers who have frequent antenatal care. The finding implies we should increase the frequency of ANC follow-up and breastfeeding counseling during ANC to increase EBF practice so as to improve maternal and child health.

The study also found that mothers who had a history of PNC are 3.77 times more likely to exclusively breastfeed their infant than their counterparts. This is in line with studies done in sub-Saharan countries [39] and studies in Ethiopia such as; Addis Ababa [36], Debremarkos [22], and Boditi [28]. This suggests providing effective postnatal care by committed health care providers can delay the introduction of breast milk substitutes/formula milk/ through exclusive breastfeeding counseling. Therefore, this counseling in post-natal care enhances EBF practice. Furthermore, mothers who had more than 36-month birth interval/spacing are 3.18 times more likely to exclusively breastfeed their infant than those mothers who had lower than three years birth interval. The possible reason might be the presence of a prolonged birth interval period leads to an optimized period of exclusive breastfeeding practice. This long birth interval might also assist the woman to get enough time to think and prepare herself for the upcoming healthy infant.

Apart from the analysis of the entire data set as a unit, separate analyses have been done for employed and unemployed mothers to appreciate the difference in factors affecting EBF practice. Accordingly, among employed mothers, having; an infant birth interval of three or more years, three or more ANC visits, and access to PNC services were more likely to exclusively breastfed their infant than their counterparts. The findings are consistent with studies done in Addis Ababa [36], and Gondar Ethiopia [19]. This finding implies strong effort should be made to increase awareness of mothers about birth spacing, ANC, and PNC service utilization to improve EBF practice so as to promote maternal and child health among employed mothers.

Similarly in the case of unemployed mothers, unemployed mothers who didn’t have breastfeeding counseling during ANC visits, are 0.15 times less likely to EBF than their counterparts. This finding is consistent with the study of Injibara [18] and Addis Ababa [36]. This might be because counseling that was given during ANC visits helps mothers to understand the advantage of EBF for their child as well as for themselves and enable them to exclusively breastfeed their child. In this study, unemployed mothers who had three or more ANC visits were five times more likely to breastfeed their infant than those mothers who had less than three ANC visits. The finding of this study is consistent with the study findings of Nepal [38], Ghana [41], Gondar [19], Injibara [18], and the Somali region [17]. The possible explanation for this result may be when unemployed mothers had frequent ANC visits; they might grasp more information about the benefits of exclusive breastfeeding practice upon potent counseling, so, this might enhance EBF practice. Similarly, Unemployed mothers who had social support from the community or organization were three times more likely to practice EBF than their counterparts. This finding is supported by the study done in Gondar [19], Hawassa [27], and the Somali region [17]. This finding suggests when women get economic, psychological, emotional, cultural, and informational support from the community or organization, the probability of EBF practice is extremely enhanced.

Unemployed mothers who had a history of breast disease are 0.28 times less likely to practice EBF than their counterparts. This finding is supported by studies conducted in Kumasi Metropolis, Ghana [34], Boditi [28], and Hawassa, Ethiopia [27]. This might be because when women had a history of breast-related problem during the lactation period, they may consider that the quality and quantity of breast milk is affected. Thus, they might have perceived breast milk as an inappropriate choice of food that affect the health of the infant.

The study only employed a quantitative method so that miss’s qualitative aspects that affect EBF practice including the attitude and beliefs of mothers related to EBF. So in the future, it is better to investigate the issue deeply by employing qualitative methods.

## Conclusion

In our study, exclusive breastfeeding practice is found lower compared to the expected standard of achievement. There is a significant difference in the prevalence of exclusive breastfeeding practice between employed and unemployed mothers. Unemployed mothers are more likely to exclusively breastfed their children than employed mothers. This showed that maternal employment is associated with low EBF practice. Some predictor variables are different for employed and unemployed mothers. The study found that birth interval, number of ANC visits, postnatal care, and employment status were significantly associated with exclusive breastfeeding among overall study participants. Among employed mothers' birth interval, the number of ANC visits and PNC service utilization is positively associated with EBF practice among employed mothers. Finally, breastfeeding counseling during ANC visits, number of ANC visits, and maternal social support from the community or organizations are significantly associated with EBF practice among unemployed mothers. Therefore Policymakers and program planners are called to come together and create a conducive environment for lactating employees including daycare centers and breastfeeding rooms. Health care workers should enhance counseling on breastfeeding practice and birth spacing for mothers during antenatal and postnatal care visits. The community should provide social support for lactating women to promote exclusive breastfeeding at home and in working areas. Generally, appropriate intervention at respective predictor variables is needed to enhance EBF practice. Furthermore, researchers are expected to dig out detail on the attitude and beliefs of mothers related to EBF.

## Data Availability

All data generated or analyzed during this study are included in this published article.

## Abbreviations

ANC: Antenatal care
AOR: Adjusted odds ratio
CI: Confidence interval
EBF: Exclusive breastfeeding
EDHS: Ethiopian Demographic Health Survey
PNC: Postnatal care
SNNPR: Southern Nation Nationalities and People
UNICEF: United Nation International Children Fund
WHO: World Health Organization
